# Benchmarking RNA-seq differential expression analysis methods using spike-in and simulation data

**DOI:** 10.1371/journal.pone.0232271

**Published:** 2020-04-30

**Authors:** Bukyung Baik, Sora Yoon, Dougu Nam

**Affiliations:** 1 Department of Biological Sciences, Ulsan National Institute of Science and Technology, Ulsan, Republic of Korea; 2 Department of Mathematical Sciences, Ulsan National Institute of Science and Technology, Ulsan, Republic of Korea; Indiana University School of Medicine, UNITED STATES

## Abstract

Benchmarking RNA-seq differential expression analysis methods using spike-in and simulated RNA-seq data has often yielded inconsistent results. The spike-in data, which were generated from the same bulk RNA sample, only represent technical variability, making the test results less reliable. We compared the performance of 12 differential expression analysis methods for RNA-seq data, including recent variants in widely used software packages, using both RNA spike-in and simulation data for negative binomial (NB) model. Performance of edgeR, DESeq2, and ROTS was particularly different between the two benchmark tests. Then, each method was tested under most extensive simulation conditions especially demonstrating the large impacts of proportion, dispersion, and balance of differentially expressed (DE) genes. DESeq2, a robust version of edgeR (edgeR.rb), voom with TMM normalization (voom.tmm) and sample weights (voom.sw) showed an overall good performance regardless of presence of outliers and proportion of DE genes. The performance of RNA-seq DE gene analysis methods substantially depended on the benchmark used. Based on the simulation results, suitable methods were suggested under various test conditions.

## Introduction

High-throughput cDNA sequencing (RNA-seq) has been commonly used for transcriptome analysis for the last decade [1]. Whereas the hybridization-based method (microarray) can only be used to measure the expression of preselected genes, RNA-seq is able to cover the whole transcriptome and has additional applications [2–8]. Even for the measurement of gene expression, RNA-seq exhibited better reproducibility and sensitivity, particularly for genes with low expression [9]. One of the main purposes of transcriptome analysis is to identify differentially expressed genes (DE genes) between different cellular conditions. Because the gene expression in RNA-seq data is represented by ‘count’ of short cDNA reads aligned to corresponding gene (or exon), discrete probabilities, such as Poisson or negative binomial (NB) distributions, have been used to model the read counts [9–13]. Based on these models, a variety of methods have been developed for differential expression analysis (DE analysis) of RNA-seq data [8, 14–16].

Several comparative studies for DE analysis methods have already been conducted to suggest suitable methods for different test conditions. Some have used real RNA-seq data to test false positive control, compared the reproducibility of DE analysis results using a reduced number of samples, and analyzed similarity of results between different methods [16–18]. Others have used benchmark data such as spike-in RNA-seq [14, 19, 20] or simulated read counts [14, 15, 21] for a more systematic comparison of false positive (or discovery) control, power, and overall discriminatory ability. Especially, simulation-based analysis enabled the comparison of methods under various test conditions related to outlier counts, sample size, proportion of DE genes, and balance of DE genes [14, 15]. Although a few methods with overall good performance have been suggested, their performance was different between studies as different benchmark datasets and test conditions were used. Moreover, new versions or variants of the widely used DE analysis packages, namely, edgeR, DESeq, and limma with improved parameter estimation or outlier treatment have been added in recent years.

In this study, (1) we compared the performance of 12 DE analysis methods using both RNA spike-in [14] and NB-based simulation data. Since the spike-in data were generated from the same bulk RNA sample (technical replicates), they exhibited much smaller dispersion estimates in NB model [22, 23] as compared to regular RNA-seq data. In addition, the proportion of DE genes in spike-in data was only 0.27%. We demonstrate that these two features of spike-in data, which are rarely observed in RNA-seq data based on biological replicates, could have caused some difference in performance between spike-in and simulation-based test results. (2) We then performed simulation analysis under various conditions using parameters estimated from RNA-seq data for biological replicates. Our tests covered the largest range of test conditions to date and demonstrated the large impact of proportion of DE genes on some DE analysis methods (e.g., edgeR and DESeq) and the robust performance of ROTS for unbalanced DE genes, in addition to confirming the dominant effect of dispersion parameter. Based on the test results, methods with a good performance were suggested for each condition. (3) Two real RNA-seq datasets were analyzed for further comparison.

## Methods

In this article, only two-sample group comparison and ‘gene’-based analysis will be considered to focus on the performance of DE analysis methods. We consider a raw read count matrix R = [*R_ij_*], *i* = 1,2,…,*n*, *j* = 1,2,…,(*m*_1_+*m*_2_) composed of non-negative integers where *n* is the number of genes analyzed, and *m*_1_ and *m*_2_ are sample sizes for the test and control groups, respectively.

### Modeling read counts using discrete probability

A read count *R_ij_* of the *i*th gene in the *j*th sample has often been modeled by discrete counting probability, such as Poisson or NB distribution. Poisson distribution is only able to address the experimental variation between replicates caused by random sampling and is appropriate for modeling the counts obtained from technical replicates [9, 10]. However, samples from different individuals (biological replicates) incorporate biological variation as well. Therefore, in biological replicate data, means are surpassed by large variances, whereas means and variances have more similar values in technical-replicate-based data.

To incorporate the increased variability in biological replicates, NB distribution has been widely used for modeling RNA-seq read counts [11–13, 24]. NB distribution has two parameters, i.e., mean *μ_ij_*≥0 and dispersion *φ_i_*≥0, and its variance is represented as $\sigma_{ij}^{2}=\mu_{ij}+\varphi_{i}\mu_{ij}^{2}$. Here, *μ_ij_* = *s_j_μ_i_*, where *s_j_* is scaling factor (or depth) of sample *j*, and *μ_i_* is the mean expression of gene *i*. If *φ_i_* is zero, NB becomes a Poisson distribution where mean and variance have the same value. Zero-inflated NB distribution was considered to model the sparse single-cell RNA-seq data [25, 26], but may not be appropriate to represent RNA-seq data. In this study, we used NB distribution to simulate RNA-seq read count data.

### DE analysis methods and comparison of their performance

We selected such DE analysis methods for RNA-seq data that exhibited relatively good performances in a previous comparative study [14–16, 18, 21]. Additionally, recent variants in widely used R packages were included. The tested methods are as follows: edgeR exact test (edgeR) [11], GLM version of edgeR (edgeR.glm) [27], robust version of edgeR (edgeR.rb) [28], quasi-likelihood edgeR method (edgeR.ql) [29, 30], robust version of edgeR.ql (edgeR.ql.rb) [31], DESeq [12], DESeq with per condition dispersion estimation (DESeq.pc), DESeq2 [32], voom with TMM (voom.tmm) [33] and quantile normalization (voom.qn) [34], voom with sample weights (voom.sw) [35, 36], baySeq [24], PoissonSeq [37], SAMseq [38], and ROTS [19]. baySeq method has been implemented with either TMM or quantile normalization (denoted as baySeq and baySeq.qn, respectively). ROTS was basically applied to voom transformed data with TMM normalization; however, ROTS was also applied to raw count data when DE genes were unbalanced between up- and down-regulated genes. A brief summary of recently developed methods as well as simulation test results of this study are described in Table 1. Detailed simulation conditions are described in ‘Simulation conditions’ section.

**Table 1:** Summary and test results of recently developed methods.

Methods	Summary	Test Results
DESeq2 [32]	Empirical shrinkage estimation of dispersions and logarithmic fold-changes. Z-test is used for DE analysis. Both outliers of dispersions and logarithmic fold-changes are treated.	DESeq2 exhibited steady and good performances regardless of outliers, sample size, proportion of DE genes, dispersions, and mean counts.
edgeR.rb [28]	Observations that deviate strongly from the model fit are given lower weights. These observation weights affect both the regression and dispersion estimates. Used when data include outlier counts.	edgeR.rb yielded more DE genes and more false positives compared to other edgeR methods. In presence of outliers and large number of samples (≥10), it exhibited outperforming AUCs.
edgeR.ql [29]	While edgeR exact test assumes the estimated dispersion is true, quasi-likelihood estimation accounts for the uncertainty of the dispersion estimates. This approach improves type I error control.	edgeR.ql showed better AUC, control of true FDR, and FPCs compared with edgeR methods, but exhibited relatively low power.
voom.qn /voom.tmm [34]	Read counts were quantile normalized (voom.qn) or normalized with TMM method (voom.tmm), and then were transformed using voom. A moderated *t*-test is used for DE analysis.	voom.tmm performed better than voom.qn except outlying sample cases. They exhibited overall good performance for most cases, but their powers were relatively low. AUC of voom.qn was noticeably decreased compared to other voom methods as the proportion of DE genes increased.
voom.sw [36]	Observations from highly variable samples are down-weighted for more accurate estimation of regression coefficients. Used when some samples have amplified dispersions	voom.sw performed like voom.tmm rather than voom.qn and showed overall good performance. When samples with amplified dispersions were included, voom.sw outperformed other methods.
ROTS [19]	voom transformation with TMM normalization and bootstrap are used for generalized *t*-statistic that maximally reproduce preselected top *k*% genes. *k* = 25 was used for our tests.	ROTS exhibited good AUC and false positive control. ROTS applied to raw count data showed slightly lowered power, but it outperformed other methods when DE genes were unbalanced and a large number of DE genes were included.

Performance of the aforementioned DE analysis methods was compared using their area under receiver operating curve (AUC), true positive rate (TPR), true false discovery rate (FDR), and false positive counts (FPCs). Here, true FDR is the proportion of non-DE genes among the significant genes and indicates the extent of reliability of predicted DE genes. We calculated true FDR only when five or more significant genes were detected in each method. FPC is the number of significant genes detected from the datasets where no DE genes were included (Type I error). Compared to the previous simulation study [15], we used a liberal threshold q-value < 0.1; this is because we applied smaller effect sizes (1.2 fold or larger) when generating DE genes. Among the compared methods, edgeR and edgeR.glm; and edgeR.ql and edgeR.ql.rb showed virtually the same results in simulation tests. DESeq.pc showed similar or better performance as compared to DESeq across all test conditions. Thus, the results of edgeR.glm, edgeR.ql.rb, and DESeq were removed to simplify the analysis. baySeq.qn results were also removed from the simulation results because the two baySeq results showed nearly the same performance in simulation tests.

### SEQC spike-in benchmark data

Sequencing Quality Control (SEQC) data pertained to the Microarray Quality Control (MAQC) study and contained replicated samples of universal human body reference RNA and human brain reference RNA with spike-in controls. We used the same count data as used in a previous DE analysis benchmark study (GEO accession GSE49712) [14] (denoted as SEQC data). SEQC data comprised two sample groups, each having five samples and 21,711 genes including 92 spike-in transcripts from External RNA Controls Consortium (ERCC). They exhibited very small dispersion estimates, which were on an average 22.5 times smaller than those of RNA-seq data of cancer samples (TCGA Kidney Renal Clear Cell dataset). We removed genes that had less than ten mean read counts and analyzed the rest 17,961 genes, which included 63 spike-in transcripts composed of 15, 16, 17, and 15 genes with 0.25, 1.5, 2, and 1 fold changes, respectively. AUCs were calculated using the 63 spike-in genes. In short, SEQC data were characterized by very small dispersion values and low proportion of DE genes (0.27%).

### Simulation conditions

The above-described DE analysis methods were tested under the following conditions:

(pDE) Different proportion of DE genes: pDE = 0.27%, 5%, 10%, 30% or 60% DE genes were included in the simulation datasets. 0.27% was considered to compare with the results for SEQC data, and 60% DE genes represent, for example, complex disease conditions such as cancer vs. normal conditions.
(SS) Sample sizes of three or ten were used in each condition.
(OL1) Random outliers: each read count could become an outlier with a probability of 0.05 or less. An outlier count was regenerated with five to ten times larger mean value.
(OL2) Low-quality sample: dispersions of 30% of samples were increased fivefold.
(OL3) Composite condition of 3 and 4: 30% of samples were generated with fivefold increased dispersions, and the 3% of read counts in the other samples were regenerated as a random outlier.
(Bal) Different balances of up and downregulated genes: Bal = 50%, 70% or 90% of DE genes were upregulated, while the remainder were downregulated.
(Disp) The same or different dispersion values between conditions were assigned for each gene.
(EF) Weak effect sizes (EF = 1.2 or larger fold changes for ten sample cases; 1.5 or larger for three sample cases) were used.

These conditions include diverse situations encountered in RNA-seq data analysis and the test results can provide a guideline for selecting an appropriate method in each situation. Our tests have the following several differences from the previous simulation study by Soneson and Delorenzi [15]:

1. Four additional edgeR methods, two additional limma methods, PoissonSeq, DESeq2, and ROTS were included in our study.
2. Wider range of DE gene proportions from 0.27% to 60% was tested, whereas only 10% and 33% DE genes were considered previously. We demonstrated that both low (0.27% or 5%) and high (60%) proportions of DE genes have substantial effects on performance of some methods (e.g., edgeR, edgeR.ql, and ROTS). The low pDE (e.g. 5%) represents the case, such as gene-knock-out experiment where only a small number of genes are expected to be differentially expressed, whereas the high pDE (e.g., 60%) represents the case of complex disease, such as cancer as compared to normal samples where the majority of genes are expected to be differentially expressed [39].
3. Effects of dispersions and mean counts on DE analysis methods were analyzed. We specifically showed that dispersions have great impacts on both the relative and absolute performances of each method.
4. In our study, weak effect sizes (1.2 or larger fold changes) were used to analyze subtle differences in performance, whereas 1.5 or larger fold changes were used previously.

### Simulation of read counts and parameter estimation

To simulate read counts, we first estimated mean and dispersion parameters for each gene from two RNA-seq datasets with different parameter distributions: TCGA Kidney Renal Clear Cell Carcinoma/normal dataset, which is available from GDAC (URL: http://gdac.broadinstitute.org) (denoted as KIRC) and inbred mouse dataset (denoted as Bottomly) [40]. KIRC is composed of independent individual samples and showed large mean counts and dispersions, whereas Bottomly is composed of samples from genetically identical mice and showed smaller means and dispersions (5.6 times and 10 times, respectively). Here, *independent samples* denote those obtained from different individuals with possibly different genetic backgrounds, such as KIRC. Independent samples typically exhibit large dispersion values [22]. We additionally built two kinds of synthetic datasets using hybrid combinations of mean and dispersion parameters obtained from KIRC and Bottomly datasets, in order to compare the effects of mean and dispersion parameters. These parameters were estimated from each of the test and control groups using edgeR package. Especially, edgeR provided the common and tagwise dispersion estimates that were used for the same and different dispersion conditions in our simulation study, respectively. All the read counts were simulated using the ‘rnbinom’ R function and estimated parameters. The inverse of the dispersion values was used for ‘size’ argument. To generate fold changes for a DE gene, we added a random number sampled from exponential distribution, exp(1) to the minimum fold changes of 1.5 (three sample case) and 1.2 (ten sample case) [15], respectively. Mean values in the test condition were multiplied or divided by these fold change values to generate up- or down-regulated DE genes, respectively. To generate outlier counts, we randomly selected approximately 5% (or less) of all read counts and regenerated those counts with five or ten times larger mean values in both test and control conditions. To generate low quality sample data, we randomly selected one sample and three samples from each condition for three and ten sample cases, respectively and regenerated those samples using five times larger dispersion values for all genes. Read counts were generated for 10,000 genes using KIRC parameters and for 5,000 genes using Bottomly or hybrid parameters. In addition, the two real RNA-seq datasets (KIRC and Bottomly) were analyzed using 12 DE analysis methods and the results were compared. All of our analyses can be reproduced using the R package which is available from https://github.com/unistbig/compareDEtools.

## Results and discussion

### Comparison of test results for SEQC spike-in and simulation data

**Fig 1:**
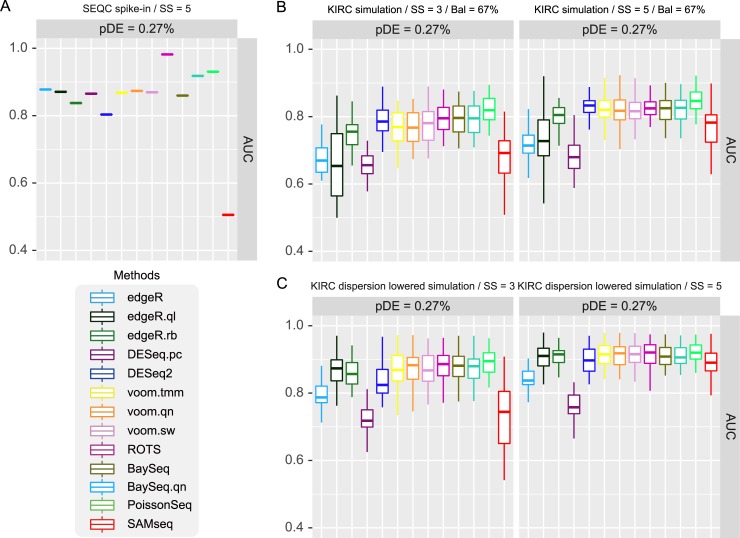
Performance comparison of 13 DE analysis methods for SEQC benchmark data and simulation data based on KIRC parameters. Three and five samples were used in simulation tests. (A) SEQC spike-in data test, 5 sample condition, 0.25, 1.5, 2 fold changes are introduced to DE genes. (B) KIRC based simulation. 1.5 or larger and 1.3 or larger fold changes are introduced to 0.27% of 10,000 genes for 3 and 5 samples, respectively. (C) Same condition as B but fixed fold changes, 0.625, 1.15, and 1.3 were introduced to DE genes. Dispersions were also lowered to SEQC level (22.5 times).

We tested the 13 DE analysis methods including two different implementations of baySeq for both, SEQC spike-in data and KIRC-based simulation data and compared their AUCs. The AUCs for SEQC data are shown in Fig 1A. ROTS outperformed other methods and was followed by PoissonSeq and baySeq.qn. SAMseq and DESeq2 showed the worst AUCs. baySeq results based on two normalizations showed a considerable difference. Then, read counts of 10,000 genes were simulated using the parameters estimated from KIRC data. Three or five samples were included in each sample group. In particular, a low pDE (0.27%) and 67% up regulated DE genes (Bal = 67%) were assumed to compare with the results for SEQC data. The results for KIRC-based simulation are shown in Fig 1B. edgeR, edgeR.ql, and DESeq.pc showed relatively low AUCs and DESeq2 showed a much better AUCs as compared to the SEQC analysis results. Such a large difference in relative performance of each method between benchmark studies confuses researchers while choosing a suitable method.

Hence, we performed further simulation tests to investigate the cause of such differences, by adjusting dispersion parameters and pDE. First, the dispersions of KIRC simulation data were decreased to the level of SEQC data (22.5 times lower) and AUCs were compared (Fig 1C). Because such small dispersions caused nearly perfect AUCs for all methods, we applied rather reduced fold changes to simulate DE genes (0.625, 1.15, and 1.3). As a result, high AUC of edgeR.ql and relatively low AUC of DESeq2 shown in SEQC results were reproduced. Then, we slightly increased pDE from 0.27% to 1% while keeping the large dispersions of KIRC (S1D Fig). In this case, only the AUC for edgeR.ql increased while DESeq2 remained the same. These results indicate that small dispersions of SEQC caused the relatively low performance of DESeq2, and both, low dispersion and low pDE of SEQC supported the high performance of edgeR.ql in SEQC result. However, the high performances of ROTS, edgeR, and DESeq.pc in SEQC analysis result were not explained by these adjustment experiments. The TPR and true FDR results are also shown in S1 Fig.

Overall, this experiment shows the extremely small dispersion and proportion of DE genes in SEQC data may not provide fully reliable evaluation of DE analysis methods and simulation tests based on parameters for biological replicates should also be considered. A similar concern of benchmarking SEQC data has also been raised when normalizing RNA-seq data [41]. Thus, we herein present the evaluation of 12 DE analysis methods using NB-based simulation under extensive test conditions.

### Simulation tests under various conditions

**Fig 2:**
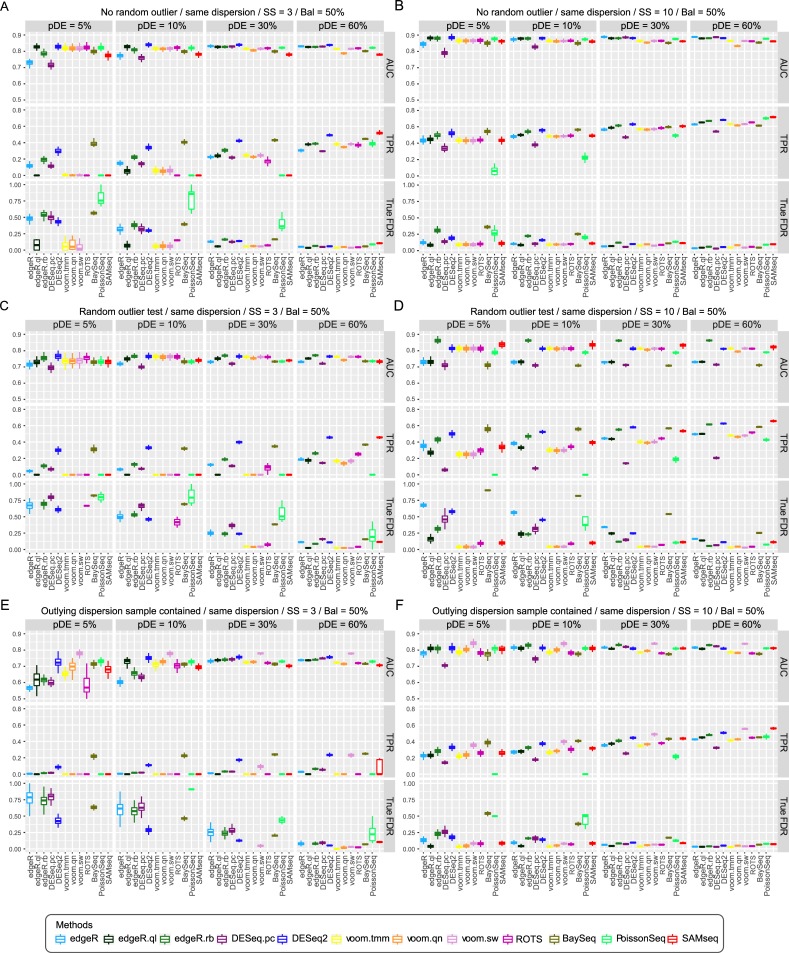
Performance comparison of 12 DE analysis methods for simulated RNA-seq data with KIRC parameters. Area under ROC curve (AUC), true positive rate (TPR) and true false discovery rate (FDR) are shown. Three and ten samples were used in each sample group for (A, C, E) and (B, D, F), respectively. (A, B) Same dispersion between test and control groups, Bal = 50%, four different proportions of DE genes (5%, 10%, 30%, and 60%) were used. (C, D) Random ourlier counts: the same condition as A-B but each read count can be a random outlier with 5% probability. (E, F) Low quality samples: the same condition as (A, B) but one and three samples of each sample group have fivefold increased dispersion parameters, respectively. True FDR graphs of some methods did not appear in low pDE condition because they detected no DE genes.

The test results are depicted as boxplots in Fig 2 and S2–S10 Figs. Basically, we compared the performance of methods based on AUCs; however, we have also considered TPRs, true FDR, and FPC to select ‘recommended’ methods under each test condition (Table 2). We attached ‘(LP)’ if a method exhibited good AUC but rather low power (TPR). If AUCs were similar, the priority was given to ‘liberal’ methods with high power and high type I error rate, considering that researchers may have additional means or information to reduce false positives. We mainly considered equal proportions of up and down regulated genes among DE genes (Bal = 50%). We further considered the unbalanced cases where 70% and 90% of DE genes were upregulated (Bal = 70% or 90%).

**Table 2:** Recommended methods for DE analysis of biological replicate RNA-seq data (simulation results).

Outlier	Sample size	%DE gene	Independent replicates (large dispersions)	Genetically identical replicates (small dispersions)
No outlier counts	3	5%	DESeq2, edgeR.ql (LP)	DESeq2 and most other methods
		≥ 30%	DESeq2, edgeR, DESeq.pc	DESeq2 and most other methods
	10	5%	DESeq2, edgeR.ql, edgeR.rb	DESeq2, edgeR methods, ROTS, PoissonSeq
		≥ 30% ^a^(Bal≥0.7)	edgeR and DESeq methods, (%ROTS)	edgeR methods, DESeq2, ROTS, (%ROTS)
With outlier counts	3	5%	DESeq2, edgeR.rb	DESeq2, ROTS
		≥ 30%	DESeq2, edgeR.rb	edgeR.rb, DESeq2, ROTS
	10	5%	edgeR.rb, SAMseq, DESeq2, voom methods, ROTS	SAMseq, DESeq2, edgeR.rb
		≥30% ^a^(Bal≥0.9)	edgeR.rb, SAMseq, DESeq2, voom methods, ROTS, (%ROTS)	edgeR.rb, DESeq2, SAMseq, (%ROTS)
With outlying samples	3	5%	Voom.sw	Voom.sw, edgeR.rb
		≥ 30%	Voom.sw, DESeq2	Voom.sw, edgeR.rb
	10	5%	Voom.sw	Voom.sw, edgeR.rb
		≥ 30%	Voom.sw, edgeR.rb	Voom.sw, edgeR.rb

^a^Under these conditions, ROTS without normalization (denoted as %ROTS) performed best. LP denotes low power.

#### Test results with large dispersions and large mean counts: Independent samples

Read counts of 10,000 genes were generated using the parameters estimated from KIRC data. Three or ten samples were generated in each sample group. To begin with, we tested for the ‘base’ condition where no outliers and no low quality samples were included, and same dispersions between sample conditions and equal proportions of up and down regulated DE genes (Bal = 50%) were assumed. Then, we further tested for alternative test conditions. Under the base condition for three samples, DESeq2 performed best (Fig 2A). Difference in the proportion of DE genes (pDE) showed dramatic changes in the edgeR and DESeq.pc test results. These two methods did not show good AUCs under low pDE condition (≤ 5%); however, they performed as well as DESeq2 under high pDE condition (≥ 30%). edgeR.ql showed considerably good AUCs, but showed very low power when three samples were used and pDE was not larger than 10%. Voom methods (voom.qn, voom.tmm, and voom.sw) exhibited similarly good AUCs in low pDE condition (5%); however, the AUC of voom.qn was lowered with the increase of pDE. A larger sample size of ten allowed every method to show better performance (Fig 2B); SAMseq, edgeR, and edgeR.rb showed large improvements in AUC.

In presence of outliers (OL1 = 5%), the best AUCs were observed for DESeq2 and edgeR.rb followed by voom.tmm, and voom.sw when three samples were used. Especially with ten samples, edgeR.rb surpassed every other method, and SAMseq was the second best. DESeq2, voom methods, and ROTS showed similarly good AUCs. We further compared DESeq2 and edgeR.rb by additionally introducing lower proportion of outliers (1% and 3%) to the base condition (S4 Fig). edgeR.rb method tended to exceed DESeq2 when pDE or sample size increased and larger proportion of outliers was introduced, and vice versa. voom.sw and voom.tmm showed similarly good performance under most test conditions. When samples with enhanced dispersions (i.e., low quality samples) were included, voom.sw outperformed other methods (Fig 2E and 2F). Under this condition, edgeR-based methods and DESeq.pc showed low AUCs for low pDE (5%) and small sample size conditions (Fig 2E). Overall, AUCs and powers tended to increase as the proportion of DE genes increased for many of the test conditions.

Next, when we applied different dispersions between conditions, the AUCs of most methods except those for edgeR, edgeR.rb, and DESeq.pc, were slightly decreased (S2 Fig). Lastly, when the fraction of upregulated DE genes was increased (Bal = 70% and 90%), the overall performance worsened (S3 Fig). The impact of this imbalance of DE genes increased as the pDE increased. When pDE was 60% and Bal = 90%, the majority of genes were up-regulated (60% * 90% = 54%), and most of the tested methods exhibited a nearly random prediction (AUC < 0.6). Interestingly, ROTS applied to raw count data *without any normalization or transformation* was much less affected by the varying proportion and imbalance of DE genes and showed outperforming AUCs when Bal ≥ 0.7 and pDE ≥ 0.3 (S3 and S7 Figs). We note that the same depth was applied to each sample in our simulation tests.

True FDRs tended to decrease as pDE increased in most cases. Voom methods, edgeR.ql, and SAMseq exhibited overall quite good control of true FDRs, while PoissonSeq and baySeq showed large true FDRs. ROTS, edgeR, and DESeq exhibited poor true FDRs for three sample case and small DE proportions (pDE ≤ 10%), but they were dramatically improved when pDE or sample size was increased. True FDRs were considerably increased as random outlier counts were introduced (Fig 2C and 2D). They were also increased by inclusion of low quality samples for KIRC case when SS = 3 (Fig 2E). True FDRs were not much affected by low quality samples when SS = 10, except for PoissonSeq (Fig 2F).

**Fig 3:**
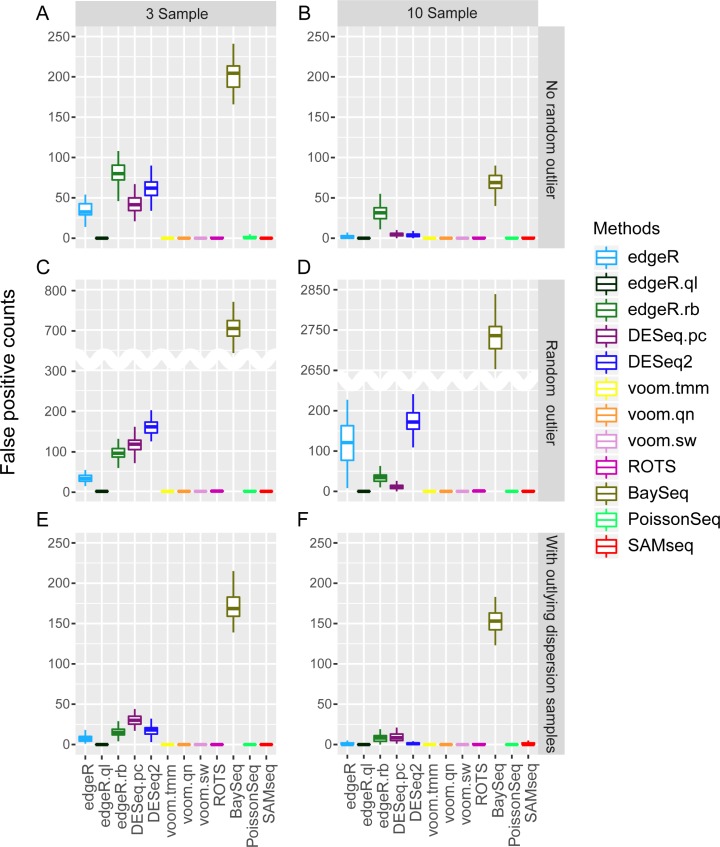
False positive count comparison of 12 DE analysis methods for simulated RNA-seq data with KIRC parameters. Three and ten samples in each sample group and three different conditions (No random outlier, random outlier, and outlying dispersion sample) were used for comparison.

We then compared the FPCs in each case by randomly sampling two groups from the same sample group (Fig 3); in other words, no DE genes were included. edgeR.ql, voom methods, PoissonSeq, and SAMseq strictly controlled FPCs, while baySeq yielded large FPCs. Although PoissonSeq exhibited very poor true FDR control, its FPC control was observed to be quite good. This shows that FPC control alone is not enough to assess the predicted DE genes, and true FDR control should also be considered. Interestingly, the two methods that account for outlier counts viz. edgeR.rb and DESeq2, did not perform better than other methods in either true FDR or FPC control (Fig 3C and 3D). They exceeded other methods in AUCs.

We also tested the composite conditions of random outliers and increased dispersions (OL3) for a large number of samples (10 and 30 samples in each condition) (S5 Fig). The overall results seemed to reflect both the results of OL1 and OL2, and edgeR.rb showed the best AUC followed by those of limma.sw and SAMseq. Interestingly, DESeq2 which performed well under both OL1 and OL2 showed a low AUC and high true FDR under this complex simulation condition.

#### Test results with small dispersions and small mean counts (genetically identical replicate case); and hybrid parameters

The same conditions were tested using the parameters estimated from Bottomly data. It is known that large mean counts and low dispersions increase the statistical power of DE analysis [21, 22]. Despite the low depths in Bottomly data, their low dispersions allowed all the methods to show much better AUCs and powers than those obtained using KIRC parameters (S6–S8 Figs). Under the base condition, many methods including DESeq2 and edgeR.rb showed similarly high AUCs; therefore, unique features of each method were not clearly distinguishable. Unlike in the KIRC case, edgeR performed as well as other edgeR-based methods when pDE was low (5%). In presence of outliers and using ten samples, the best performing methods were edgeR.rb, DESeq2, and SAMseq followed by voom based methods. True FDRs for Bottomly were overall much improved as compared to those for KIRC, and their patterns were overall similar to each other.

To compare the impacts of mean and dispersion parameters, we additionally created simulation data with hybrid parameters by combining KIRC means and Bottomly dispersions (denoted as mKdB) and Bottomly means and KIRC dispersions (denoted as mBdK), respectively. As shown in S10 and S11 Figs, all 12 methods showed high powers for low dispersion datasets (mKdB and Bottomly), while they showed relatively low powers for high dispersion datasets (mBdK and KIRC). In contrast, mean had only limited effects; performance for mKdB dataset was only slightly better than that for Bottomly dataset, and the performances for mBdK and KIRC datasets were observed to be nearly the same.

#### A guide for choosing methods

We have shown through simulation tests that dispersion values, proportion of DE genes, and outlier counts largely impact the performance of each method. However, it is not always clear which condition in Table 2 corresponds to the data at hand except for the sample size. We have previously demonstrated that RNA-seq data generated from independent samples tended to exhibit large dispersion values (approximately, ≥ 0.1), whereas those obtained from genetically identical replicates yielded relatively small dispersion values (approximately, 0.01–0.1) [22]. Thus, the replicate type used is able to roughly suggest the distribution of dispersions. To be more precise, the researcher may also use existing packages, such as edgeR, directly to estimate dispersion values from their own read count data.

For outlier counts, DESeq2 automatically truncates them or simply remove genes that contained a potential outlier count depending on the sample size. However, for edgeR package, the user has to choose a method between edgeR and its robust version. If the given data are highly noisy, edgeR.rb may be most effective. For moderately noisy data, both DESeq2 and edgeR.rb perform better than their original methods. Recently developed methods can help detect outlier counts in RNA-seq data [42, 43].

Lastly, the proportion of DE genes are not known to the user. However, we can roughly estimate the proportion from the test condition. In complex disease, such as cancer, the majority of genes are differentially expressed compared to the normal condition, where the condition ≥30% can be considered. If a single gene with potentially limited effects was knocked out, much less proportion of DE genes are expected compared to complex disease conditions. In ambiguous cases, several DE analysis methods can be tested together to roughly estimate DE genes from significantly detected genes.

### Real RNA-seq data analysis

We also compared the 12 DE analysis methods by analyzing the two RNA-seq datasets (KIRC and Bottomly) that exhibited different distribution of parameters. As the true DE genes are not known for real data, we only compared the number of significantly detected genes and FPCs. KIRC contained 72 normal and 72 cancer samples. Bottomly contained ten C57BL/6J strain and eleven DBA/2J strain samples. Three, five, ten, and twenty (KIRC only) sample sizes were considered. After removing the genes with average read count of less than ten, 16,621 genes and 8,550 genes remained for KIRC and Bottomly, respectively. We used q-value < 0.1 to select DE genes. To compare FPCs, we used only the normal sample group for KIRC, where two contrasting groups were randomly sampled for DE analysis. The same analysis was done for the DBA/2J sample group of Bottomly. Each experiment was repeated fifty times and the results are represented as boxplots in Fig 3A and 3B.

**Fig 4:**
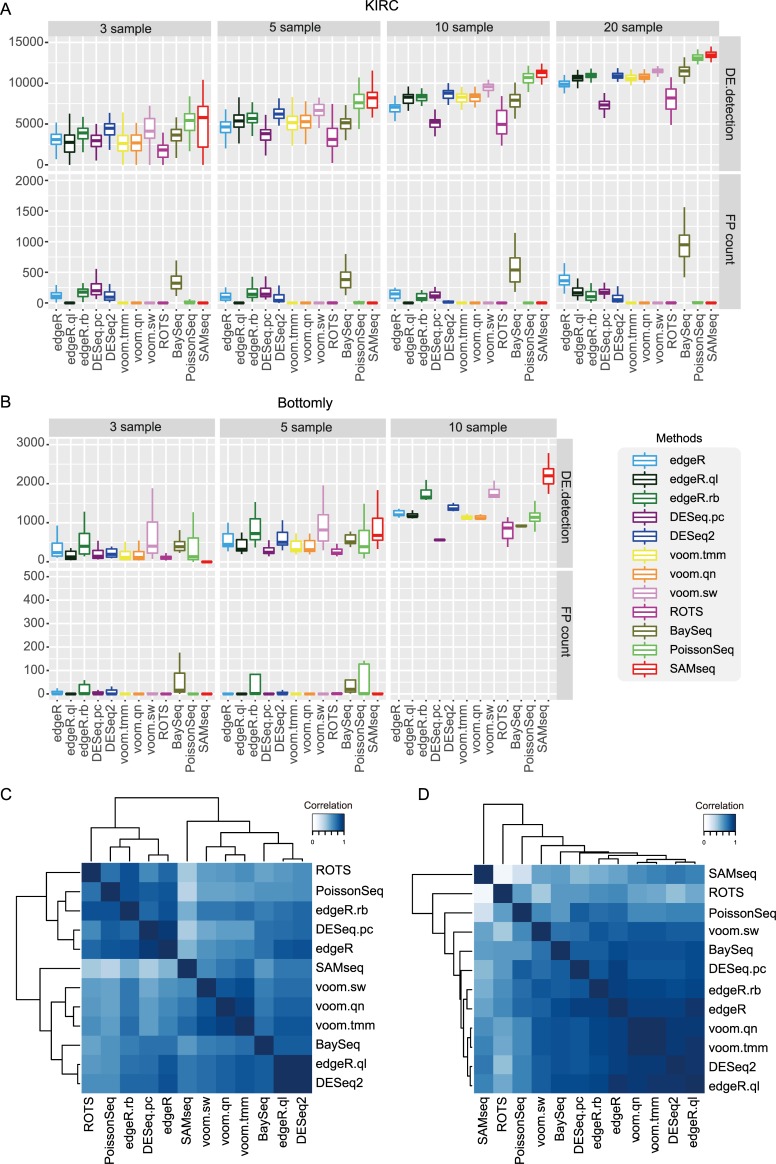
Analysis on KIRC and Bottomly. (A, B) The number of DE genes between two sample groups and FP counts under varying sample sizes. (A) DE genes between normal and cancer sample groups and 3 to 20 sample size conditions were tested for KIRC. (B) DE genes between C57BL/6J strain and DBA/2J strain sample groups and 3 to 10 sample size conditions were tested for Bottomly. (C, D) Hierarchical clustering of DE genes obtained from 12 DE analysis methods based on spearman rank correlation. Top 5000 and 500 DE genes are used to calculate spearman rank correlation for KIRC and Bottomly datasets, respectively.

Most results showed similar patterns between the two datasets except that PoissonSeq detected relatively small number of DE genes for Bottomly. SAMseq and voom.sw detected the largest numbers of DE genes, while DESeq.pc and ROTS detected the smallest numbers of DE genes for both datasets. We assumed that KIRC included many DE genes between cancer and normal conditions (e.g., pDE = 60%), and found high similarity between the number of detected DE genes (Fig 4A; 3 and 10 samples) and the TPR of simulation results (Fig 2A and 2B; pDE = 60%); we may expect such a similarity though DE genes and TPR represent different concepts. We also found a similarity in FPCs in that edgeR.ql, voom methods, ROTS, PoissonSeq, and SAMseq exhibited very small or no FPCs while baySeq exhibited relatively large FPCs in both datasets.

Additionally, we compared the similarity of DE analysis results between the 12 methods. Five samples were randomly selected from each sample group, and DE analysis was repeated fifty times using each method. Based on our observation that most methods detected approximately 5,000 and 500 DE genes with five samples for KIRC and Bottomly, respectively (Fig 4A and 4B), the top 5,000 and 500 DE genes in each method were selected based on DE *q*-values for KIRC and Bottomly, respectively. The selected DE genes obtained using each method were pooled and sorted by their average DE *q*-values. Then, Spearman rank correlations between each pair of methods were calculated. The similarity matrix as well as clustering results for KIRC and Bottomly are shown in Fig 4C and 4D, respectively. In both cases, edgeR.ql and DESeq2 were closely combined. Voom methods were also closely combined to each other. However, voom.sw was separated from other voom methods in Bottomly, thus implying the presence of outlying samples in Bottomly. Indeed, principal component analysis for the two datasets (S12 Fig) showed samples in KIRC exhibited a relatively homogeneous and compact distribution in each condition, while those in Bottomly showed a rather heterogeneous distribution and less clear difference between conditions. ROTS was most distantly clustered from other methods in both cases. Similar clustering patterns were reproduced when we used top 3,000 and 300 genes in KIRC and Bottomly, respectively (S13 Fig).

### Comparison of the computing time

**Fig 5:**
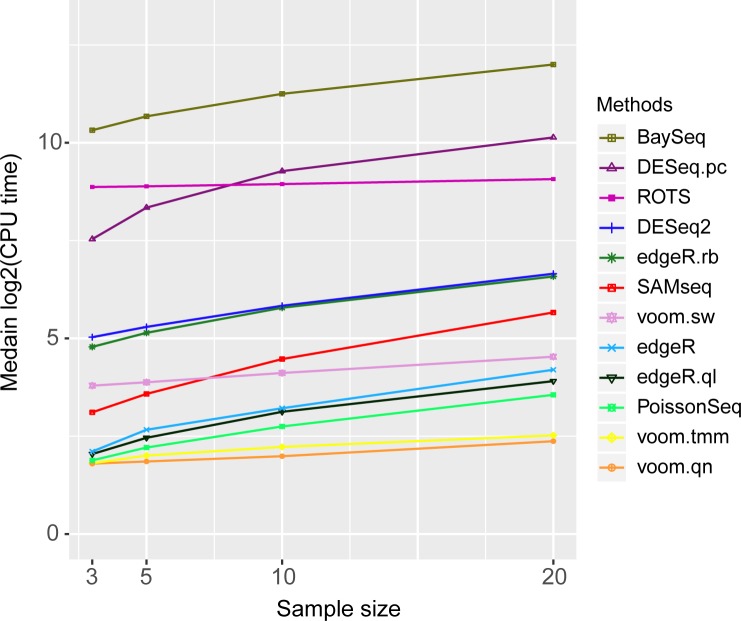
CPU-times to run DE analysis methods for KIRC data. Each method was run 10 times for different sample sizes 3, 5, 10, and 20.

Lastly, the running times for each method for analyzing KIRC data were compared (Fig 5). Each method was executed ten times using ‘proc.time’ R function. A subset of KIRC containing 16,621 genes with 3, 5, 10, and 20 samples were used for this measurement. All the methods were run on a Linux machine with Inter X5670 hexa core processor and 20Gb of 1333MHz DDR3 memory. The two fastest methods were voom.qn and voom.tmm followed by PoissonSeq and edgeR.ql. Most methods finished computing within 100 cpu-time. The slowest was observed to be baySeq (1,000 to 4,000 cpu-time) followed by DESeq.pc and ROTS. As sample size increased, many methods including baySeq, DESeq.pc, DESeq2, edgeR, and SAMseq showed a linear increase in the computing time.

## Conclusion

We compared the performances of DE analysis methods including recent developments available from popularly used packages, for RNA spike-in, simulated read counts, and real RNA-seq data. We demonstrated the extremely small dispersions and proportion of DE genes in the spike-in data could cause quite different benchmarking results. We performed most extensive simulation tests based on NB model and obtained many new or more detailed results in addition to confirming previously tested results. Our test results suggest appropriate methods in each experimental condition.

## Supporting information

S1 Fig Performance comparison of 13 DE analysis methods for SEQC benchmark and simulation data. Area under ROC curve (AUC), true positive rate (TPR) and true false discovery rate (true FDR) are shown. Three and five samples were used in simulation tests. (A) Test results for SEQC spike-in data (5 samples). 0.25, 1.5, and 2 fold changes were introduced to DE genes. (B) KIRC based simulation. Not less than 1.5 or 1.3 fold changes are introduced to 0.27% of 10,000 genes for 3 and 5 samples, respectively. (C) Two conditions are different from B: fixed fold changes of 0.625, 1.15, and 1.3 were introduced to DE genes, and dispersions were also lowered to SEQC level (22.5 times) (D) The same condition as A but DE proportion was increased to 1%.
(EPS)

S2 Fig Simulation study with KIRC parameters. Dispersions between test and control groups are different (A) Three samples per each group and four different proportions of DE genes (5%, 10%, 30%, and 60%) were used. (B) Sample size is increased to ten. (C, D) Each read count can be a random outlier with 5% probability.
(EPS)

S3 Fig Simulation study with KIRC parameters for imbalanced DE proportions where 70% or 90% DE genes are upregulated (Bal = 70% and 90%). No random outlier was included, and same dispersions were used between conditions. (A) Three samples per each group and Bal = 70% were used for four different proportions DE genes (5%, 10%, 30%, and 60%). (B) Bal = 90%. (C, D) The same condition as A-B, but sample size is increased to ten.
(EPS)

S4 Fig Simulation study for low probabilities (1% and 3%) of each read count being an outlier (KIRC parameters). (A) 1% probability for each read count to be an outlier was applied. Three samples per each group and four different proportions DE genes (5%, 10%, 30%, and 60%) were used. (B) The same condition as A, but the sample size is increased to ten. (C, D) The same as A and B, but the outlier probability of 3% is used.
(EPS)

S5 Fig Simulation test with KIRC parameters for complex conditions. 30% of each sample group have fivefold increased dispersion parameters and 3% of counts in the other samples were regenerated as outliers. Ten and 30 samples were used in each sample group for (A) and (B), respectively.
(EPS)

S6 Fig Performance comparison of 12 DE analysis methods for simulated RNA-seq data with Bottomly parameters. Area under ROC curve (AUC), true positive rate (TPR) and true false discovery rate (true FDR) are shown. Three and ten samples were used in each sample group for (A, C, E) and (B, D, F), respectively. (A, B) Same dispersion between test and control groups, Bal = 50%, four different proportions DE genes (5%, 10%, 30%, and 60%) were used. (C, D) Random outlier counts: the same condition as A-B but each read count can be a random outlier with 5% probability. (E, F) Low quality samples: the same condition as (A, B) but one and three samples of each sample group have fivefold increased dispersion parameters.
(EPS)

S7 Fig Simulation study with Bottomly parameters. Dispersions between test and control groups are different (A) Three samples per each group and four different proportions of DE genes (5%, 10%, 30%, and 60%) were used. (B) The sample size is increased to ten. (C, D) The same condition as A-B but each read count can be a random outlier with 5% probability.
(EPS)

S8 Fig Simulation study with Bottomly parameters for imbalanced DE proportions where 70% or 90% DE genes are upregulated (Bal = 70% and 90%). Same dispersions were used between conditions. (A) Three samples per each group and Bal = 70% were used for four different proportions of DE genes (5%, 10%, 30%, and 60%). (B) Bal = 90%. (C, D) Sample size is increased to ten.
(EPS)

S9 Fig False positive count comparison of 12 DE analysis methods for simulated RNA-seq data with Bottomly parameters. Three and ten samples were used in each sample group for (A, C, E) and (B, D, F), respectively. (A, B) Same dispersions between test and control groups are assumed. (C, D) Random outlier counts: the same condition as A-B but each read count can be a random outlier with 5% probability. (E, F) Low quality samples: the same condition as (A, B) but one and three samples of each sample group have fivefold increased dispersion parameters.
(EPS)

S10 Fig Simulation study with hybrid parameters (mBdK: Means of Bottomly and dispersions of KIRC). Same dispersions between test and control groups are used. (A) Three samples per each group and four different proportions of DE genes (5%, 10%, 30%, and 60%) were used. (B) Sample size is increased to ten. (C, D) Random outlier counts: the same condition as A-B, but each read count can be a random outlier with 5% probability.
(EPS)

S11 Fig Simulation study with hybrid parameters (mKdB: Means of KIRC and dispersions of Bottomly). Same dispersions between test and control groups are used. (A) Three samples per each group and four different proportions of DE genes (5%, 10%, 30%, and 60%) were used. (B) Sample size is increased to ten. (C, D) Random outlier counts: the same condition as A-B, but each read count can be a random outlier with 5% probability.
(EPS)

S12 Fig Principal Component Analysis for (A) KIRC and (B) Bottomly datasets.
(EPS)

S13 Fig Similarity of DE analysis methods. Hierarchical clustering of DE genes based on spearman rank correlation. The union of top 3000 and 300 DE genes obtained from 12 DE analysis methods were clustered between different methods based on spearman rank correlation for (A) KIRC and (B) Bottomly datasets, respectively.
(EPS)

## List of abbreviations

DE: differentially expressed
NB: negative binomial
SEQC: Sequencing Quality Control
AUC: area under receiver operating curve
TPR: true positive rate
FDR: false discovery rate
FPC: false positive count
